# Multiple-ASSR Interactions in Adults with Sensorineural Hearing Loss

**DOI:** 10.1155/2012/802715

**Published:** 2012-09-25

**Authors:** Ieda M. Ishida, David R. Stapells

**Affiliations:** School of Audiology & Speech Sciences, The University of British Columbia, Friedman Building, Room 443, 2177 Wesbrook Mall, Vancouver, BC, Canada V6T 1Z3

## Abstract

The multiple auditory steady-state response (multiple-ASSR) technique, where thresholds for up to 8 frequencies (4 in each ear) are obtained simultaneously, is currently of great interest for audiometric assessment of infants. Although threshold estimates using the multiple-ASSR appear to be reasonably accurate, it is not currently known whether it is more efficient to use multiple stimuli or single stimuli when testing individuals with sensorineural hearing loss (SNHL). The current study investigated the effect of single versus multiple simultaneous stimuli on the 80- and 40-Hz ASSRs in adults with normal hearing or SNHL. Results showed significant interactions (i.e., decreased amplitudes) for both ASSRs going from single to multiple stimuli in one ear. Going from multiple one ear to multiple two ears did not further reduce the amplitude of the 80-Hz ASSR. At the 40-Hz rate, however, there was a further amplitude decrease going from one-ear multiple to two-ear multiple stimuli. Importantly, these interactions did not differ between the normal-hearing and SNHL groups. Although supportive of the multiple-ASSR technique, there are likely situations where it is more efficient to use single stimuli. Future studies are required to assess these interactions in infants with varying degrees and configurations of hearing loss.

## 1. Introduction

Auditory steady-state responses (ASSRs) have received much recent attention by clinicians and researchers for reviews, see [1, 2]. ASSRs to stimuli modulated in the 70–110 Hz range (the “80-Hz” ASSR) have their generators primarily in auditory brainstem structures; in the 35–45 Hz range (the “40-Hz” ASSR), ASSR sources are primarily cortical in origin with brainstem contributions [3]. 

Most recent attention has focussed on the 80-Hz ASSR, which will likely be recommended for routine clinical assessment of auditory threshold in young infants in the near future, possibly in place of the tone-evoked auditory brainstem response (ABR), which is the current gold-standard technique [4–6]. However, lack of appropriate normative and clinical data in infants currently limits the clinical use of the 80-Hz ASSR [5, 7, 8]. Research in the 1980s indicated the 40-Hz ASSR is difficult to record in infants [9, 10]; however, many studies have indicated the 40-Hz ASSR can provide accurate estimates of thresholds in adults, for example, [11].

 Similar to tone-ABR recordings, the ASSR may be recorded to single-frequency stimuli presented to one ear at a time. This is the “single-stimulus” ASSR method, for which many clinical data have been published, for reviews, see [5, 12, 13]. Alternatively, it is also possible to record ASSRs to multiple stimuli (i.e., several frequencies) presented simultaneously to one or both ears. Although clinical data for this technique are more limited (for reviews, see [5, 12, 13]), this “multiple” ASSR technique has the potential to gather more information in a shorter amount of time, thus speeding up test time [14–16]. If there are no interactions between responses when multiple stimuli are presented (i.e., amplitudes are not smaller when stimuli are presented together), then the time to record responses is simply reduced by the number of stimuli presented simultaneously [17]. Even if some amplitude reductions (“interactions”) exist, the presentation of multiple simultaneous stimuli may still be more efficient than the presentation of a single stimulus provided that the reduction in amplitude is less than $1/\sqrt{K}$, where *K* is the number of stimuli presented at the same time [14, 18]. If ASSR amplitudes are smaller due to interactions, more sweeps must be averaged to reduce the EEG noise sufficiently to detect these smaller responses. Because EEG noise decreases predictably by the square root of the number of sweeps averaged, we can use this to determine whether the increased number of sweeps required to detect a smaller multiple-ASSR amplitude is offset by the gain in information [16]. With multiple stimuli, *K* times the information (e.g., the number of frequencies) in a given sweep is obtained compared to the single-stimulus technique, thus provided we do not need to average more sweeps than a factor of $\sqrt{K}$, the multiple-ASSR will be more efficient. For example, if ASSR amplitudes decrease by 50% due to interactions when recording responses to eight simultaneous stimuli (e.g., four frequencies in each ear), the presentation of multiple stimuli is still more efficient provided the ASSR amplitude at the frequency of interest is greater than 35% $\left(1/\sqrt{K}\right)$ of the single-stimulus ASSR amplitude [18].

 In adults, the presentation of multiple stimuli simultaneously does not cause a decrease in 80-Hz ASSR amplitude when stimuli are presented at 60 dB SPL or less in one or both ears, provided that the carrier frequencies are separated by at least an octave [14, 15, 19]. However, for 75–80 dB SPL stimuli, statistically significant interactions between responses to stimuli are seen such that amplitudes in the 4-stimulus multiple condition (4 frequencies to one ear) decrease to 52–58% of their amplitude in the single-stimulus condition [14, 20, 21], with no further decrease in the 8-stimulus multiple condition (4 frequencies to each ear) [21]. Despite this, when compared to the single-stimulus technique, the 80 Hz multiple-ASSR technique remains more efficient, at least in adults [14, 21]. In contrast to adults, in young infants, the presentation of 60 dB SPL multiple stimuli simultaneously results in a significant decrease in 80-Hz ASSR amplitude. The reasons for this are uncertain; however, the decreases are not enough to make the multiple-ASSR technique less efficient, at least for normal infants [18]. 

 As with the 80-Hz ASSR, the 40-Hz ASSR shows no interactions when multiple stimuli are presented at low (30 dBHL) intensities [22]. However, in contrast to the 80-Hz ASSR, the 40-Hz ASSR shows significant interactions when multiple stimuli are presented at 60 dB SPL, decreasing to approximately 60% of their amplitude in the single-stimulus condition [14, 19, 22]. In further contrast to the 80-Hz ASSR, the 40-Hz ASSR decreases by an additional 33% when multiple stimuli is presented to both ears dichotically [14, 22]. For higher stimulus intensities (75–80 dB SPL), 40-Hz ASSR shows interactions similar to those for the 80-Hz ASSR except that only the 40 Hz shows an additional decrease with dichotic stimulation [14, 22]. Taking into account these amplitude reductions, the use of multiple stimuli for the 40-Hz ASSR may only be more efficient when testing low and moderate intensities (no. 60 dB SPL) and only for single-ear stimulation [22].

 Many studies have shown reasonably accurate estimates of behavioural thresholds using the 80- and 40-Hz ASSRs to multiplestimuli for reviews, see [5, 11, 12, 23], and multiple-stimulus ASSR systems are currently being marketed to clinicians for threshold assessment. However, no study has assessed single- versus multiple-stimulus ASSR interactions in individuals with sensorineural hearing loss (SNHL); thus, it is not known if the multiple-stimulus ASSR technique is more efficient compared to the single-stimulus ASSR when hearing loss is present. It is reasonable to hypothesize that there would be greater interactions when cochlear hearing loss is present due to broader cochlear filters [24]. If the multiple-stimulus ASSR interactions are too large, it may be more efficient to present single rather than multiple stimuli when assessing individuals with SNHL. The present study assessed multiple-ASSR interactions by recording 80- and 40-Hz ASSRs to single versus multiple stimuli in adult groups with either normal hearing or SNHL. 

## 2. Methods

### 2.1. Participants and Procedure

A total of 24 adults participated in this study: 12 with normal hearing aged between 23 and 63 years (mean age 38.2 ± 13.0 yrs; 6 female) and 12 with SNHL aged between 22 and 69 years (mean age 52.3 ± 15.9 yrs; 7 female). The normal-hearing group had pure-tone behavioural thresholds of 20 dB HL (ANSI, S3.6-1996) or better in both ears for 500, 1000, 2000, and 4000 Hz. Their average (and standard deviation, SD) hearing thresholds were 8 (6.2), 5 (6.6), 5 (6.6), and 7 (8.1) dB HL, respectively for 500, 1000, 2000, and 4000 Hz in the test ear. The participants with SNHL had pure-tone behavioural thresholds for their test ear greater than 20 dB HL for at least one of 500, 1000, 2000, and 4000 Hz, with bone-conduction thresholds within 5 dB of thresholds for air-conduction stimuli (i.e., no conductive or mixed hearing loss). However, because ASSR stimuli were presented at 80 dB HL, participants with SNHL had to have a threshold of 65 dB HL or better for at least one of 1000 or 2000 Hz in the test ear. Mean (SD) test-ear thresholds for the SNHL group were: 30 (15), 36 (16), 47 (12), and 53 (16) dB HL for 500, 1000, 2000, and 4000 Hz respectively. Mean (SD) thresholds for their nontest ear were 33 (19), 44 (25), 50 (22), and 59 (23) dB HL.  Table 1 shows the test-ear hearing thresholds for individual SNHL participants. 

 Subject eligibility criteria excluded participants with any of the following: (i) age greater than 70 years; (ii) external/middle ear involvement (e.g., ear infection) in either ear; (iii) neurological involvement (e.g., cerebral palsy, multiple sclerosis); (iii) 80- or 40-Hz ASSR absent in monotic single conditions for either 1000 or 2000 Hz. (As responses in the MS condition typically show the largest amplitudes, this criterion ensured (i) the ability to assess the effects of going from single to multiple stimuli and (ii) results for all subjects were available for all conditions. Two additional subjects were excluded due to this criterion.) Finally, (iv) subjects were rejected (i.e., considered “noisy”) if two ASSR recordings out of three reached the limit of 60 sweeps before reaching noise criteria (see below for description of the noise criteria). There were three additional subjects rejected for “noisy” results and therefore not included in the data for calculation of final results.

 Informed written consent, approved by The University of British Columbia Behavioural Research Ethics Board, was obtained before commencing the study and the participants were paid an honorarium at the end of the session. Testing was performed in a double-walled sound-attenuating booth. The session began with pure-tone behavioural audiometry for air- and bone-conduction stimuli. 40-Hz ASSR results were then obtained, with the subjects typically watching a muted DVD movie with subtitles, followed by the recording of the 80-Hz ASSR with the subject reclined in a comfortable chair and instructed to relax or sleep. Four conditions were recorded: (i) dichotic multiple (DM): both ears simultaneously, all four frequencies (500, 1000, 2000, and 4000 Hz); (ii) monotic multiple: test ear only, all four frequencies simultaneously; (iii) monotic single for 1000 Hz: test ear only; (iv) Monotic Single for 2000 Hz: test ear only. The recording order of the conditions was randomized. The test ear was chosen according to the following criteria: (i) randomized, if symmetrical hearing loss or (ii) for asymmetrical hearing loss, the ear with smaller difference between 1000 and 2000 Hz thresholds.

### 2.2. ASSR Stimulus Parameters

The stimuli for the 80- and 40-Hz ASSR were generated by the Rotman MultiMASTER research system [25] with carrier frequencies of 500, 1000, 2000, and 4000 Hz. Using a digital-to-analog conversion rate of 31,250 Hz [26], stimuli were sinusoidally amplitude (100%) and frequency (25%) modulated (i.e., AM/FM stimuli), for the 80-Hz ASSR at 77.15, 84.96, 92.77, and 100.59 Hz (left ear) and 81.06, 88.87, 96.68, and 105.47 (right ear), for 500, 1000, 2000, and 4000, respectively. Modulation rates for the 40-Hz ASSR were 35.94, 39.06, 42.19, and 45.31 Hz (left ear) and 37.5, 40.62, 43.75, and 46.87 Hz (right ear). The modulation rates were chosen to ensure each EEG recording contained an integer number of modulation cycles and each recording sweep contained an integral number of carrier frequencies [25].

 The stimuli were calibrated using a Larson Davis model 824 sound-level meter (using “peak SPL” minus 3 dB to determine peak-to-peak equivalent SPL). The four AM/FM tones were each calibrated separately in dB HL and then combined. The intensity of the individual stimuli was kept constant at 80 dB HL for all conditions tested. The stimuli were presented via air conduction through ER-3A insert earphones in both ears. Both earphones were kept on throughout the recording session.

### 2.3. ASSR Recording Parameters

ASSRs were recorded using the Rotman MultiMASTER research system. Three gold-plated electrodes were used to record the electrophysiologic responses at (i) Fz (noninverting), (ii) midline at the nape (inverting), and (iii) left mastoid (ground). All inter-electrode impedances were kept below 3 kOhms at 10 Hz. The EEG was amplified 80,000 times and filtered using a bandpass of 30 to 250 Hz (12 dB/octave) for the 80-Hz ASSR and a bandpass of 5 to 100 Hz for the 40-Hz ASSR [11]. A 1250 Hz analog-to-digital conversion rate was used [27]. Each EEG recording sweep lasted 13.107 seconds and was comprised of 16 epochs of 1024 data points each (0.8192 seconds per epoch). Artifact rejection was set to eliminate epochs of electrophysiological activity with amplitudes exceeding ±60 *μ*V for the 80-Hz ASSR and ±80 *μ*V for the 40-Hz ASSR.

 ASSRs were averaged in the time domain and then analyzed online into the frequency domain using a fast Fourier transform (FFT). In order to decrease the effect of EEG noise, weighted averaging (80-Hz ASSR: 70–110; 40-Hz ASSR: 30–50 Hz) was used, for further details, see [28]. The *F*-ratio was calculated by MultiMASTER and estimated the probability that the amplitude of the ASSR at the modulation frequency for each carrier frequency was significantly different from the amplitude of the background noise in adjacent frequencies within ±60 bins of the modulation frequency (“mean noise”) [25]. Recordings were automatically stopped if significant responses were not reached within 60 sweeps. Recordings always continued for a minimum of 12 sweeps and continued until the noise criteria were met for all carrier frequencies (test ear). If the mean noise after a maximum of 60 sweeps did not drop below the noise criteria of 16.5 nV for the 80-Hz ASSR [21] and 33 nV for the 40-Hz ASSR [11, 13, 20, 22], the condition was repeated (see above for subject eligibility criteria). Although not a stopping criterion, response significance (*P* < .05) was also noted.

### 2.4. Statistical Analyses

In addition to descriptive statistics (mean, SD, etc.), amplitudes were analyzed, separately for the 80- and 40-Hz ASSRs, using a mixed-model analysis of variance (ANOVA) (1 between-subjects factor (2 groups), 2 within-subjects factors (3 conditions × 2 stimulus frequencies)). To compare results between 80- and 40-Hz ASSRs, amplitudes were normalized to a percentage of the amplitude in the MS condition and assessed using a mixed-model analysis of variance (ANOVA) (1 between-subjects factor (2 groups), 3 within-subjects factors (2 rates, 2 conditions, 2 stimulus frequencies)).

Differences in the above mixed-model ANOVAs were considered significant if *P* < .05. Newman-Keuls post hoc comparisons were performed for significant (*P* < .05) main effects and interactions.

Percent-amplitude results allowed us to assess the efficiency of the single versus multiple conditions. Even if significant decreases in ASSR amplitudes with presentation of multiple stimuli are seen, this does not necessarily mean the multiple-stimulus condition is less efficient than the single-stimulus condition. Relative efficiency is a measure that considers the increase in information relative to the decrease in amplitude when going from single- to multiple-stimulus conditions. Provided the reduction in amplitude is less than $1/\sqrt{K},$ where *K* is the number of stimuli presented simultaneously, the multiple stimulus condition remains more efficient [14, 18, 22]. (For a full description of relative efficiency, see Hatton and Stapells [18].) Thus, the 4-stimulus MM condition is more efficient than the MS condition provided its amplitude is more than 50% of the MS condition's amplitude. Similarly, the 8-stimulus DM condition is more efficient if its amplitude is more than 35% of that of the MS condition. Relative efficiency (RE) was calculated using the following formula: RE = (AMPm/AMPs)${}^{*}\sqrt{K}$, where RE is the relative efficiency; AMPmis the individual subject's amplitude for specific multiple condition; AMPs is the individual subject's amplitude for same frequency in the MS condition; *K*  is the number of simultaneous stimuli. 

 Multiple-stimulus conditions with RE values >1 are more efficient than the MS condition; those with RE values <1 are less efficient. In the present study, dependent-sample *t*-tests were carried out to determine whether RE results in a multiple-stimulus condition were significantly different from the corresponding MS condition, where RE always equals “1". After Bonferroni correction for multiple *t*-tests, results were considered significant if *P* < .00625 (alpha level of .05 divided by 8 tests = .00625).

## 3. Results

**Figure 1:**
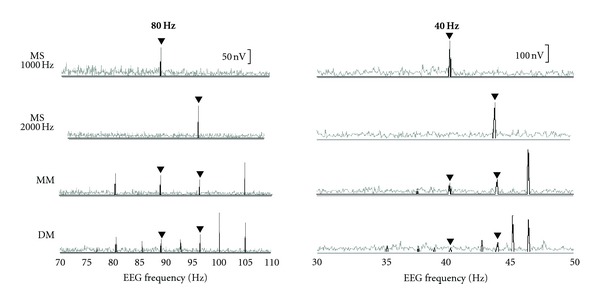
Representative normal-hearing subject's ASSR amplitude spectra in the monotic single (MS), monotic multiple (MM), and dichotic multiple (DM) stimulus conditions. Filled triangles indicate response to 1000 and 2000 Hz stimuli.

**Figure 2:**
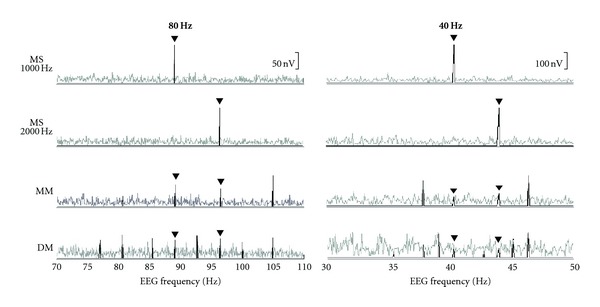
Representative SNHL subject's ASSR amplitude spectra in the MS, MM, and DM stimulus conditions. Filled triangles indicate response to 1000- and 2000-Hz stimuli.

Typical results for a normal-hearing subject and a subject with SNHL are shown in Figures 1 and 2, respectively. Across all conditions, 40-Hz ASSR amplitudes are much larger than 80 Hz amplitudes for both subjects. The effects of single versus multiple stimuli, however, differ between the two rates. For both subjects, going from single (MS) to multiple (MM and DM) stimuli results in a decrease in 80-Hz ASSR amplitude. However, going from multiple one ear (MM) to multiple two ears (DM) does not further reduce the amplitude of the 80-Hz ASSR. In contrast, at the 40 Hz rate, in addition to a large amplitude decrease going from single (MS) to multiple one ear (MM), going to one-ear multiple to two-ear multiple (DM) results in a further decrease in amplitude. It is this latter decrease that is particularly different between 80- and 40-Hz ASSRs.

### 3.1. 80-Hz ASSR Amplitudes

**Figure 3:**
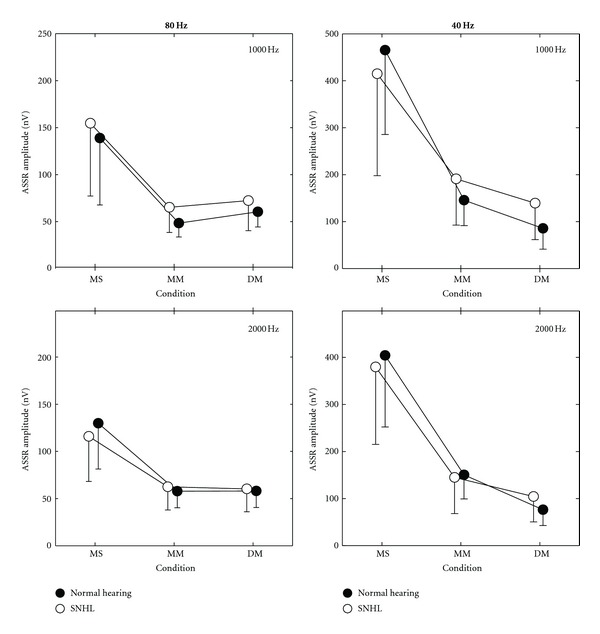
Mean (and SD) ASSR response amplitudes for both groups for the MS, MM, and DM conditions at the 40 and 80 Hz rates.

Figure 3 shows the mean ASSR amplitudes for the three conditions (MS, MM, DM), for both groups (normal hearing and the SNHL), for both frequencies (1000 and 2000 Hz), and for both rates (80 and 40 Hz).

As can be seen in the left panels of Figure 3, there were no differences in the 80 Hz response amplitudes for the two groups, with a mixed-model ANOVA revealing no significant main effects or interactions involving “group” (group: *P* = .651; condition × group: *P* = .757; frequency × group: *P* = .072; condition × frequency × group: *P* = .404). The frequency × group trend (*P* = .072) was due to the larger amplitude (albeit not significant) for 1000 Hz (97.5 nV) compared to 2000 Hz (79.6 nV) in the SNHL group; this difference was not seen in the normal-hearing group (1000 Hz: 82.6 nV; 2000 Hz: 81.9 nV).

 As would be expected from previous studies of the effects of single versus multiple stimuli presented at higher intensities [14, 19, 20, 29], Figure 3 shows that multiple stimuli resulted in significant reduction in the amplitudes of the 80-Hz ASSR. The above ANOVA showed a significant main effect of condition (*P* < .001). Post hoc analysis revealed that the amplitudes in the MM and DM conditions were significantly smaller (*P* < .001) than those of the MS condition, with no difference between the MM and the DM conditions (*P* = .501). Across all conditions, there was no significant main effect of frequency (*P* = .054). However, as indicated by a significant condition × frequency interaction (*P* = .015), the 80-Hz ASSR amplitude for 1000 Hz was significantly larger than the 2000 Hz amplitude in the MS condition (146.8 versus 122.9 nV; *P* < .001); this difference disappeared in the multiple conditions (*P* = .202–.306). 

### 3.2. 40-Hz ASSR Amplitudes

As can be seen in the right panels of Figure 3, there were no differences in the 40-Hz ASSR amplitudes between the two groups, with a mixed-model ANOVA revealing no significant main effect of group (*P* = .880) and no significant interactions involving group (*P* = .312–.641). 

 Similar to previous studies on the 40-Hz ASSR [14, 20, 22, 30] of the effects of single versus multiple stimuli, the right panel of Figure 3 shows that multiple simultaneous stimuli resulted in significant reductions in the amplitudes of the 40-Hz ASSR. In a mixed-model ANOVA with the same design as that for the 80-Hz ASSR results, a significant main effect for condition (*P* < .001) was found, such that 40-Hz ASSR amplitude in the MS condition (416.0 nV, pooled across groups and frequencies) was significantly (*P* < .001) reduced to 153.5 nV in the MM condition, with a further significant (*P* = .048) amplitude reduction in the DM condition (101.5 nV). There were no significant interactions involving condition (*P* = .294–.381).

As would be expected from the literature for review, see [2], response amplitudes for the 40-Hz ASSR were significantly larger for 1000 Hz (237.7 nV) compared to 2000 Hz (209.6 nV; main effect of frequency: *P* = .042). There were no significant interactions involving frequency (*P* = .340–.641).

### 3.3. Comparison of the 80 and 40 Hz Results Change in Amplitude from MS to the MM and DM Conditions

As previous studies have shown for review, see [2] and also obvious in Figure 3, the 40-Hz ASSR amplitudes are 2 to 3 times larger than those of the 80-Hz ASSR. To compare results between both rates, amplitudes were normalized to a percentage of the amplitude in the MS condition.  Table 2 shows the percent amplitude results, as well as the relative efficiency results, for the MM and DM conditions. 

 Overall, a mixed-model ANOVA showed a significant rate main effect (*P* = .003), with 40-Hz ASSR percent amplitude being smaller (80 Hz: 49.3%; 40 Hz: 36.4%), indicating a greater impact of multiple simultaneous stimuli on the 40-Hz ASSR. The exception to this was for the SNHL group at 1000 Hz, where the 40-Hz ASSR percent amplitude was the same as the 80 Hz (rate × frequency × group interaction: *P* = .032). Across both groups, rates, and frequencies, the DM condition shows significantly smaller percent amplitudes than the MM (MM: 45.3%, DM: 40.4%; condition main effect: *P* = .049). However, the smaller percentage for DM was due to a significant decrease in the 40-Hz ASSR percent amplitude; no change occurred in the 80-Hz ASSR percent amplitude (rate × condition interaction: *P* < .001). Thus, 40-Hz ASSR amplitude decreases further with 2-ear multiple stimuli whereas 80-Hz ASSR shows no additional decrease; both rates showed similar percent decreases in the MM condition relative to the MS condition (rate × condition interaction: *P* < .001). With the exception noted above (SNHL 40-Hz ASSR percent amplitude at 1000 Hz), there were no significant main effects or interactions involving groups or stimulus frequency.

 As the results in Table 2 indicate, there are many amplitude means (in % of MS amplitude) that are smaller than 50% in the 4-stimulus MM condition. To be more efficient, however, amplitude should not decrease more than 50% [1/$\sqrt{4}$ = 0.5]. The “efficiency” of a test is quantified by the calculation of “relative efficiency (RE),” the mean values of which are also shown in Table 2. Across both groups, the MM condition shows RE values less than or close to one (0.84–1.03) for the 80-Hz ASSR, suggesting single stimuli would be more (or at least, equally) efficient than the MM condition. However, 80-Hz ASSR results for the 8-stimulus DM condition show RE values well above one (~1.5) indicating DM is more efficient than both one-stimulus (MS) or four-stimulus (MM) conditions. For the 40-Hz ASSR, similar RE results were seen for the MM condition; however, the large decrease in amplitude for 8-stimulus 2-ear DM condition results in lower RE values (0.71–0.92). One oddity, however, were the relatively high 40-Hz ASSR percent amplitudes and, thus, high RE values seen in MM (1.26) and DM (1.27) conditions for the SNHL group's 1000-Hz results.

 Dependent sample *t*-tests were carried out to determine whether RE results in a multiple condition (MM or DM) were significantly different from the single-stimulus condition (MS). The 80-Hz ASSR DM condition was significantly more efficient at both 1000 (*P* < .001) and 2000 Hz (*P* < .001) than the corresponding MS condition. In contrast, the 40-Hz ASSR DM condition at 2000 Hz was significantly less efficient (*P* = .001) than the corresponding MS condition. The five other comparisons indicated RE values for MM or DM conditions were not significantly different (*P* = .021–728) from the MS single condition. 

## 4. Discussion

 The current study investigated the effect of single versus multiple simultaneous stimuli on the 80- and 40-Hz ASSRs in individuals with normal hearing or SNHL. Results show significant interactions (i.e., decreased amplitude) for both 80- and 40-Hz ASSRs going from single (MS) to multiple (MM) stimuli in one ear. Going from multiple one ear (MM) to multiple two ears (DM) does not further reduce the amplitude of the 80-Hz ASSR, whereas at the 40 Hz rate, there is a further amplitude decrease going from one-ear multiple (MM) to two-ear multiple (DM) stimuli. These interactions were the same in both normal-hearing and SNHL groups.

 The finding of similar interactions between the normal-hearing and SNHL groups was not expected. Because individuals with SNHL have broader cochlear filters, we hypothesized the SNHL group would show larger amplitude decreases with multiple stimuli (i.e., more interactions). This result could be due to the use of the 80 dB HL stimulus intensity, which would result in relatively broad cochlear activation for both groups, and thus, interaction would be similar. It is not possible to test lower intensities (where interactions would be smaller) in individuals with SNHL as such levels would be subthreshold. (Although the SNHL group was, on average, 14 years older than the normal-hearing group, this cannot explain the lack of difference between groups. First, the age difference is not large enough to affect the ASSR, and second, if anything, one would expect smaller 80-Hz ASSR amplitudes in the older subjects [31], which, although not significant, was the opposite to the present study's results.) Although the current study's results may suggest that interactions are not a greater concern in individuals with SNHL, further research is needed to investigate the interactions at other frequencies and intensities. 

 The finding of significant interactions for the 80-Hz ASSR to multiple stimuli (MM and DM) for higher intensity stimuli (75–80 dBHL) is consistent with previous studies [14, 20, 21, 29]. The source of the interactions for the 80-Hz ASSRs is not yet known but reflects cochlear and/or brainstem mechanisms [3, 17]. Clinically, these interactions increase the number of averages required to reach given signal-to-noise ratio. This increased time may be offset by obtaining responses to multiple stimuli simultaneously. The relative efficiency measure provides a quantification of this trade off. In the current study, the relative efficiency of the 80-Hz ASSR for the one-ear, four-stimuli, multiple-stimulus condition (MM) was not significantly different from the single-stimulus (MS) condition. In contrast, testing both ears simultaneously with four stimuli (DM) was significantly more efficient than MS. From this, one might conclude that the multiple-stimulus 80-Hz ASSR is more efficient than the MS condition providing two ears are tested simultaneously. However, the multiple-ASSR may not be more efficient when significant differences between ears and/or frequencies exist, in which case perhaps it might be best to do single stimuli especially if only one ear is being tested at a time.

 The interaction for the 40-Hz ASSR for single versus multiple stimuli in one ear is such that relative efficiency values for the MM condition are not significantly better than the MS condition, similar to the 80-Hz ASSR. In contrast to the 80-Hz ASSR, however, relative efficiency values for the multiple two-ear (DM) condition are significantly lower than the MS condition for the 40-Hz ASSR, at least for high intensities; therefore, it does not appear that multiple simultaneous stimuli are more efficient for the 40-Hz ASSR. At lower intensities (30 and 55 dBHL), however, previous research showed that both the MM and DM conditions are more efficient than the MS condition for the 40-Hz ASSR, at least for normal-hearing subjects [22]. Similar to the 80-Hz ASSR, however, the 40-Hz ASSR to multiple stimuli may not be more efficient when significant differences between ears and/or frequencies exist.

 The finding of smaller 40-Hz ASSR interactions for the SNHL group at 1000 Hz may reflect either (i) a cochlear phenomenon, whereby there is a decreased influence of 2000 Hz on the 1000-Hz responses due to greater hearing loss at 2000 Hz, and/or (ii) a cortical phenomenon, with plasticity-related enhancement of function in the cortical areas representing 1000 Hz due to the hearing loss present at 2000 Hz. There are many reports in the literature indicating cortical plasticity enhancing responses to frequencies at the edge of sloping hearing loss for review, see [32]. The fact that the 80-Hz ASSR, which reflects cochlear and brainstem processes, did not show this pattern suggests the relatively smaller interactions for the 40-Hz ASSR at 1000 Hz for the SNHL group do not originate from cochlear processes.

 Audiological use of the 40-Hz ASSR is of particular interest for threshold estimation in adults undergoing assessment for medicolegal and/or compensation purposes. The 80-Hz ASSR is smaller in amplitude, for review, see [2], and requires significantly longer test times [11]. Although most studies have focused on the 40-Hz ASSR to single stimuli, the multiple-stimulus technique has also shown excellent threshold estimation using the 40-Hz ASSR in adults with hearing loss [11]. Results of the present study suggest that when using multiple stimuli for the 40-Hz ASSR, each ear should be tested separately (i.e., MM). Because the 40-Hz ASSR is difficult to record in sleeping infants [9, 10, 33], clinical applications of the ASSR in infants have focused on the 80-Hz ASSR. The results of the present study suggest two-ear testing, that is, (DM) would be more efficient for 80-Hz ASSR. 

 There are several limitations to the present study. First, the stimulus intensity was limited to 80 dBHL. Lower intensities (e.g., 60 dBHL) would have made it difficult to test individuals with hearing loss. Testing at higher intensities (e.g., 90–100 dBHL) would allow a greater range of hearing losses, but also entail issues concerning loudness discomfort and possibly risk of additional hearing loss due to overstimulation. A second limitation is the relatively narrow range of degrees and configurations of hearing loss in the SNHL group, with most subjects having mild-to-moderate slightly sloping hearing loss. In order to have tested more significant hearing loss, we would have had to use a stimulus intensity higher than 80 dBHL to ensure we get a response in at least the MS condition. A third limitation is we only looked at interactions for 1000 and 2000 Hz stimuli. A primary reason why we restricted the study to these frequencies was that the additional test frequencies (e.g., 500 and/or 4000 Hz) would significantly increase the test time for each subject. Further, testing at 4000 Hz would have required a higher intensity due to the subjects' sloping hearing loss. A fourth limitation is that this study assessed interactions in adults with hearing loss; however, the group of primary interest for clinical testing is infants with hearing loss. 

## 5. Conclusions 

 The current study shows that ASSR interactions with 80 dBHL stimuli, which result in smaller amplitudes when going from single to multiple stimuli, do not differ between adults with normal hearing and SNHL. This suggests that the multiple-ASSR technique is not less efficient in subjects with SNHL compared to those with normal hearing. Previous research has suggested the multiple-stimulus 80-Hz ASSR technique is more efficient than the single-stimulus ASSR in normal infants [18]. Although supportive of the multiple-ASSR technique, there are likely situations where it is more efficient to use single stimuli. Future studies are required to assess these interactions in infants with varying degrees and configurations of hearing loss. 

## Figures and Tables

**Table 1:** Pure-tone behavioural hearing thresholds (test ear, in dBHL) of the SNHL group.

Subj. no.	500 Hz	1000 Hz	2000 Hz	4000 Hz
SNHL1	30	35	45	35
SNHL2	10	20	25	30
SNHL3	25	40	40	40
SNHL4	40	55	60	45
SNHL5	35	30	35	60
SNHL6	65	65	65	65
SNHL7	25	45	45	60
SNHL8	35	50	60	90
SNHL9	20	35	50	50
SNHL10	20	20	35	40
SNHL11	15	10	50	60
SNHL12	40	30	50	60

Mean	30	36	47	53
SD	15	16	12	16

SD: standard deviation.

**Table 2:** Percent amplitude and relative efficiency.

		80 Hz				40 Hz			
		1 kHz		2 kHz		1 kHz		2 kHz	
		MM	DM	MM	DM	MM	DM	MM	DM
Normal hearing	% Amp (M)	40.3	53.33	45.94	46.44	33.46	19.94	43.22	21.55
	SD	15.33	25.80	7.69	9.83	12.57	10.54	26.89	10.59
	RE (M)	0.81	1.51	0.92	1.31	0.67	0.56	0.86	0.61
	SD	0.31	0.73	0.15	0.28	0.25	0.30	0.54	0.30

SNHL	% Amp (M)	44.47	50.99	60.86	57.24	63.20	45.07	39.19	28.83
	SD	17.35	18.10	26.48	25.94	53.74	34.89	14.33	15.51
	RE (M)	0.89	1.44	1.22	1.62	1.26	1.27	0.78	0.82
	SD	0.35	0.51	0.53	0.73	1.07	0.99	0.29	0.44

ALL	% Amp (M)	42.11	52.16	51.29	51.84	47.04	32.50	40.92	25.19
	SD	15.65	21.83	18.61	19.96	41.12	28.29	21.07	13.51
	RE (M)	0.84	1.48	1.03	1.47	0.94	0.92	0.82	0.71
	SD	0.31	0.62	0.37	0.56	0.82	0.80	0.42	0.38

Percent amplitude and RE calculated relative to each subject's results in the MS condition.

M: mean; SD: standard deviation; RE: relative efficiency.
